# 498. Covid-19 Hospitalization Rates in Kidney Transplant Patients Treated with Tixagevimab/Cilgavimab

**DOI:** 10.1093/ofid/ofad500.567

**Published:** 2023-11-27

**Authors:** Katherine Dugan, Jillian Casale, Heather DeSanto, Colleen Dowling, Steven Smoke

**Affiliations:** Cooperman Barnabas Medical Center, Livingston, New Jersey; Cooperman Barnabas Medical Center, Livingston, New Jersey; Cooperman Barnabas Medical Center, Livingston, New Jersey; Cooperman Barnabas Medical Center, Livingston, New Jersey; Cooperman Barnabas Medical Center, Livingston, New Jersey

## Abstract

**Background:**

Tixagevimab/cilgavimab (T/C) is a monoclonal antibody that was previously authorized for pre-exposure prophylaxis of COVID-19 in individuals with moderate to severe immune compromise. Limited evidence of safety and efficacy in immunocompromised populations was available at the time of authorization. The purpose of this study is to assess the safety and efficacy of T/C for pre-exposure prophylaxis of COVID-19 in a large cohort of kidney transplant patients.

**Methods:**

This was a single-center Institutional Review Board-approved retrospective study. This study included adult patients that received a kidney transplant within the prior two years and received T/C between January 1, 2022, and March 31, 2022. The efficacy population comprised those that received the full 300 mg / 300 mg dose and the safety outcome population included those that received any amount of T/C. Patient demographics, transplant course history, and clinical interventions required during hospitalization due to COVID-19 infection were collected. The primary efficacy outcome was the rate of hospitalization for COVID-19 infection within 6 months of T/C administration. The secondary safety outcome was the rate of serious adverse events. Causality was assessed with the Naranjo algorithm.

**Results:**

A total of 240 patients were reviewed for eligibility and 122 were included. A majority (60%) were male, 34% white and the median age was 57 (IQR 47-62) years old. The efficacy population included 115 patients. Most patients had hypertension (96%), decreased renal function (median CrCl 48 mL/min [33-63]), and had been vaccinated for COVID-19 (98%). The rate of hospitalization for COVID-19 infection within 6 months of T/C was 2% (2). The median time to hospitalization was 158 days. Neither patient required an ICU admission, mechanical ventilation, or experienced mortality. There was no incidence of heart failure, myocardial infarction, or anaphylaxis secondary to T/C. Other adverse effects (2%) considered possibly due to T/C included cardiac arrest (1) and atrial fibrillation (1).

**Conclusion:**

Rates of COVID-19 hospitalization and adverse events within 6 months of T/C were low within a large cohort of kidney transplant patients.

**Disclosures:**

**All Authors**: No reported disclosures

